# A Sensor for Broken Wire Detection of Steel Wire Ropes Based on the Magnetic Concentrating Principle

**DOI:** 10.3390/s19173763

**Published:** 2019-08-30

**Authors:** Yiqing Zhang, Luyang Jing, Weixiao Xu, Weixia Zhan, Jiwen Tan

**Affiliations:** College of Mechanical and Automotive Engineering, Qingdao University of Technology, Qingdao 266520, China

**Keywords:** magnetic concentration, magnetic flux leakage, finite element method, broken wire, steel wire rope

## Abstract

Electromagnetic testing is the most widely used technique for the inspection of steel wire ropes. As one of the electromagnetic detecting approaches, the magnetic flux leakage (MFL) method has the best effect for the detection of broken wires. However, existing sensors based on MFL method still have some problems. (1) The size of the permanent magnet exciter is usually designed according to experience or rough calculation, and there is not enough depth analysis for its excitation performance; (2) Since the detectable angular range for a single Hall component is limited, Hall sensor arrays are often employed in the design of MFL sensors, which will increase the complexity of the subsequent signal processing due to the extensive use of Hall components; (3) Although the new magneto-resistance sensor has higher sensitivity, it is difficult to be applied in practice because of the requirement of the micron-level lift-off. To solve these problems, a sensor for the detection of broken wires of steel wire ropes based on the principle of magnetic concentration is developed. A circumferential multi-circuit permanent magnet exciter (CMPME) is employed to magnetize the wire rope to saturation. The traditional Hall sensor array is replaced by a magnetic concentrator to collect MFL. The structural parameters of the CMPME are optimized and the performance of the magnetic concentrator is analyzed by the finite element method. Finally, the effectiveness of the designed sensor is verified by wire breaking experiment. 1–5 external broken wires, handcrafted on the wire rope with a diameter of 24 mm, can be clearly identified, which shows great potential for the inspection of steel wire ropes.

## 1. Introduction

Steel wire ropes have important applications in mine lifting, cable-stayed bridges, metallurgy, elevators, and so on. They are widely used due to their high strength, light weight, reliability, and efficiency [1]. Since wire ropes usually work in harsh environments, although they suffer from a variety of types of damage such as broken wire and wear, which affects the safety of production and even threatens the lives of workers [2]. To avoid accidents, manual inspection and regular replacement are generally used in engineering. However, manual inspection is time-consuming and laborious, and regular replacement usually causes great economic waste. According to a survey, more than 70% of replaced wire ropes still have initial breaking strength [3]. Therefore, it is of great importance to develop scientific and effective devices to inspect steel wire ropes.

**Figure 1:**
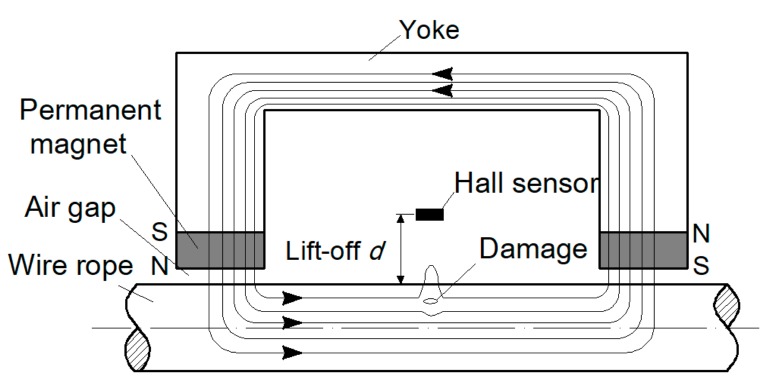
Schematic of the MFL method.

There are two types of defects of steel wire ropes, loss of metallic cross-sectional area (LMA) and a localized fault (LF), and broken wire is the most typical outcome of LF. Among the various nondestructive testing techniques, magnetic flux leakage (MFL) method is most economical and effective for broken wire detection [4,5,6,7,8]. The basic principle of the MFL method is shown in Figure 1, where the permanent magnet magnetizes part of the wire rope to saturation, and a closed magnetic circuit is formed between the wire rope, the magnet and the yoke. When no damage is present, most of the magnetic induction lines pass through the inside of the wire rope. When there is a damage such as broken wire, the magnetic resistance of the damaged position increases, and part of the magnetic induction line leaks out to form the MFL. Magnetic sensitive elements are placed between the poles of the magnet to sense the MFL signal. The condition of the wire rope can be determined according to the received signal.

For decades, many experts and scholars have done a lot of research on the design of damage detecting sensors based on the MFL method [9,10,11,12,13]. Cao Y.N. et al. [9] proposed an approach for detecting LF of steel wire ropes using an annular array of Hall components. A back propagation (BP) network is used to classify the faults. This method can differentiate the degree and the width of local defects. Zhang J. et al. [10] applied the giant magneto-resistance (GMR) sensor to the detection of LF and LMA of the wire rope. Through the use of compressed sensing wavelet filtering and BP neural network, the accuracy and reliability of MFL sensor is improved. Wu B. [13] designed an MFL sensor based on tunnel magneto-resistive device. A blind hole with dimension of 0.3 mm in both depth and diameter is detectable for the sensor. The axial resolution to two adjacent notches with a width of 0.2 mm of the TMR-based MFL sensor can be less than 2.5 mm. However, arranging annular arrays of Hall components undoubtedly increases the complexity of the signal processing. Using magneto-resistive sensors can improve the sensitivity of the sensor, but it is difficult to be applied to actual inspections due to the micron-level requirements of the lift-off [13]. Therefore, designing a sensor that can be applied to the detection in actual engineering and is both simple and effective, has always been a problem for the condition monitoring of wire ropes.

The magnetic concentrating detection technology provides a new direction for the development of MFL sensors. The detection of wire ropes usually requires the arrangement of a plurality of magnetic sensitive elements. Especially for the large diameter wire ropes, it usually needs dozens of magnetic sensors, which greatly increases the difficulty of signal processing in the later stage. The magnetic concentrating principle can realize the leak-free detection of large diameter wire ropes through a small number of magnetic sensitive elements [14,15]. Kang et al. [14] theoretically analyzed the feasibility of magnetic concentrating detection. It is proved by calculation that the magnetic concentrator can collect the MFL and guide it into the Hall component through the bridge between the concentrators to realize the collection of weak leakage flux. At the same time, it can eliminate the strand-waveform noise of wire ropes and improve signal-to-noise ratio of the MFL signal. Wang et al. [15] analyzed the performance of the magnetic concentrators on collecting the MFL by finite element simulation and proposed the structure which is suitable for collecting the magnetic leakage flux. The structure was verified by experiments, which further promoted the development of the magnetic concentrating detection.

In this study, a sensor, which is constructed of ring-shaped magnets, a yoke, and a magnetic concentrator, is designed to detect broken wires of steel wire ropes. We optimized the structural parameters of the circumferential multi-circuit permanent magnet exciter (CMPME) and analyzed the performance of the magnetic concentrator on collecting MFL through the finite element method. Finally, the proposed sensor is applied in an experiment for broken wire detection. The induced MFL signal can be clearly recognized and the signal-to-noise ratio of the MFL signal is improved by discrete wavelet transform (DWT).

The rest of the paper is organized as follows. Section 2 describes the design principle of the magnetic circuit of the CMPME and the basic theory of magnetic concentrating detection. In Section 3, the structure parameters of the exciter are optimized by simulation, and the performance of the magnetic concentrator on collecting MFL is analyzed. Section 4 illustrates the experimental settings, steps and result analysis. Finally, the conclusions are drawn in Section 5.

## 2. Theoretical Background

### 2.1. Structural Design Principle of CMPME

**Figure 2:**
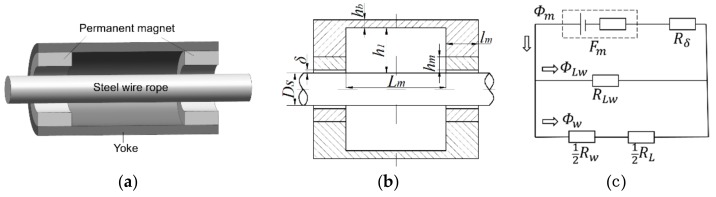
Schematic of the CMPME: (**a**) Three-dimensional structure; (**b**) Two-dimensional structure; (**c**) Equivalent simplified magnetic circuit.

The circumferential multi-circuit permanent magnet exciter (CMPME) is to magnetize the wire rope to saturation through a ring-shaped magnet. The magnetization direction of the ring magnet is radial. This structure can make the excitation effect more uniform, and the wire rope can become more easily magnetized to saturation. The three-dimensional structure of CMPME is shown in Figure 2a, and the two-dimensional structure is displayed in Figure 2b. Its equivalent simplified magnetic circuit [16] in the absence of damage to the wire rope is shown in Figure 2c.

In Figure 2c,. $F_{m}$. represents the magnetomotive force of the magnet; $R_{\delta }$ is the magnetic resistance of the air gap between the magnet and the surface of the wire rope; $R_{LW}$ denotes the air gap reluctance between the inner and outer end faces of the yoke and the surface of the wire rope; $R_{W}$ is the magnetic resistance of the magnetized wire rope, and $R_{L}$ is the magnetic resistance of the yoke; $\Phi_{m}$ is the magnetic flux in the magnet; $\Phi_{LW}$ denotes the magnetic flux of the air gap between the yoke and the surface of the wire rope; $\Phi_{W}$ is the magnetic flux inside the wire rope. The weak magnetic flux leaking into the air at the magnetic pole is ignored here. According to the Kirchhoff’s law for a magnetic circuit, the relationship between the magnetic parameters is presented as follows:

(1) $$\Phi_{m}=\Phi_{LW}+\Phi_{w}$$

(2) $$\left\{\begin{array}{l} R_{LW}\Phi_{LW}=H_{m}h_{m}-R_{\delta }\Phi_{m}\\ R_{LW}\Phi_{LW}-\frac{1}{2}(R_{W}+R_{L})\Phi_{W}=0 \end{array}\right.$$

For the convenience of calculation, the magnetic conductance *G* is introduced. *G* is defined as follows:

(3) $$G=\frac{1}{R}$$

Determining the Equations of the magnetic conductance of each section of magnetic circuit by analytic method, which are presented as follows:

(4) $$G_{\delta }=\frac{2\pi \mu_{0}l_{m}}{\ln\left[\left(D_{mw}+2\delta \right)/D_{mw}\right]}$$

(5) $$G_{W}=\mu_{rw}\pi D_{mw}^{2}/\left(4L_{m}\right)$$

(6) $$G_{L}=\mu_{rL}\pi h_{b}\left(D_{mw}+2h_{1}+h_{b}\right)/L_{m}$$

(7) $$\begin{array}{l} G_{LW}=4\mu_{0}\left[\frac{D_{mw}}{2}+\sqrt{(\delta +h_{m})h_{1}}ln\left(\frac{h_{1}}{\delta +h_{m}}\right)\right]\\ +4\mu_{0}\left[\frac{D_{mw}}{2}+\sqrt{(\delta +h_{m})\left(h_{1}+h_{b}\right)}ln\left(\frac{h_{1}+h_{b}}{\delta +h_{m}}\right)\right] \end{array}$$

where $G_{\delta }$, $G_{W}$, $G_{L}$, $G_{LW}$ are the magnetic conductance corresponding to each magnetic resistive. $D_{mw}$ is the calculated diameter of the steel bar equal to the effective metal area of the wire rope. As shown in Figure 2b, $l_{m}$ is the length of the permanent magnet along the axial direction of the wire rope; δ denotes the air gap between the permanent magnet and the surface of the wire rope; $h_{m}$ represents the radial thickness of the magnet; $L_{m}$ is the distance between the two magnetic poles; $h_{1}$ denotes the radial gap between the inner side of the yoke and the surface of the wire rope; $h_{b}$ is the wall thickness of yoke. According to the magnetic flux density that the wire rope needs to be magnetized, the parameters of the permanent magnet exciter can be calculated by the Equations (2)–(7), then optimized by the simulation and experimental results, and finally the size of the exciter is determined.

### 2.2. Principle of Magnetic Concentrating Detection

**Figure 3:**
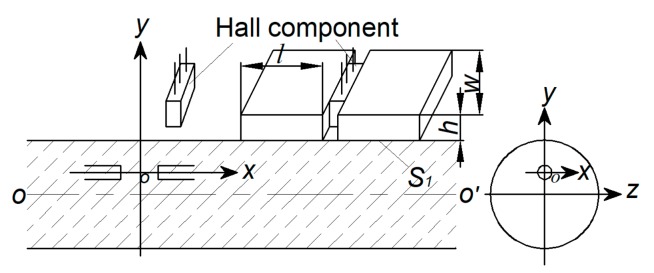
Schematic of magnetic concentrating detection.

The magnetic concentrating detection is realized by magnetic concentrators made of high magnetic permeability materials. The best detecting effect can be achieved by determining the suitable shape and size of the concentrator. Figure 3 is the schematic diagram [14].

As can be seen from Figure 3, a single Hall sensor collects a component of the spatial magnetic leakage flux, and its output Hall potential is calculated by Equation (8):

(8) $$\begin{array}{l} V_{H}=K_{H}I_{C}\iint_{S_{H}}B_{Z}(x,y,z)d_{x}d_{y}\\ =K_{H}I_{C}\Phi_{H}(x,y,z) \end{array}$$

where $K_{H}$ is a constant, determined by the characteristics of the Hall component; $I_{C}$ is the current flowed in the Hall component, and $S_{H}$ is the sensing area of the Hall component. Therefore, the Hall potential is directly related to the sensing area of the Hall component, which is related to the magnetic flux $\Phi_{H}\left(x,y,z\right)$.

Magnetic concentrators are added on both sides of the sensing surface of the Hall component, and its size is *l* × *w* × *h*. The magnetic flux $\Phi_{1}$ between the two concentrator is calculated as follows:

(9) $$F_{1}(x,y,z)=\iint_{S_{1}}B_{Z}(x,y,z)d_{y}d_{z}$$

The collecting effect of magnetic field at both ends of the magnetizer is ignored here. In the Equation (9), $S_{1}$ is the side faces area of the two concentrator parallel to the xoz plane. If the magnetic flux $\Phi_{1}$ is completely imported into the Hall component, since $S_{1}$ is larger than $S_{H}$, the Hall potential will increase accordingly, which will increase the strength of the MFL signal in turn.

## 3. Finite Element Analysis of the Sensor Structure

As one of the most popular numerical methods at present, the finite element method (FEM) has been widely used in magnetic field simulation [17,18,19]. In this paper, the structure parameters of CMPME are investigated and the performance of magnetic concentrator on collecting magnetic leakage flux is analyzed through FEM.

### 3.1. Simulation of Main Parameters of CMPME

The CMPME in this paper is designed according to the wire rope with a diameter of 24 mm. The parameters of the exciter to be determined are shown in Figure 2b. The air gap δ and the axial distance $L_{m}$ are the most important parameters affecting the excitation effect [20,21]. It can be known from Equation (4) that, with other parameters unchanged, the magnetic conductance $G_{\delta }$. will decrease along with an increase in air gap δ. According to Equation (3), the magneto-resistance $R_{\delta }$ will increase, which will cause more magnetic potential drop. Therefore the magnetic energy cannot be effectively utilized to the magnetization of the wire rope. However, too small air gap is not conducive for the wire rope to pass through the sensor during the detection process. In order to minimize the magnetic potential drop and taking into account the traversability of the wire rope, δ = 2 mm is optimal for the sensor design. According to the principle of light weight, the radial thickness of the magnet $h_{m}$ = 10 mm and the wall thickness of yoke $h_{b}$ = 5 mm. Due to the requirement of locating detecting elements in the exciter, $h_{1}$ = 30 mm is chosen.

**Figure 4:**
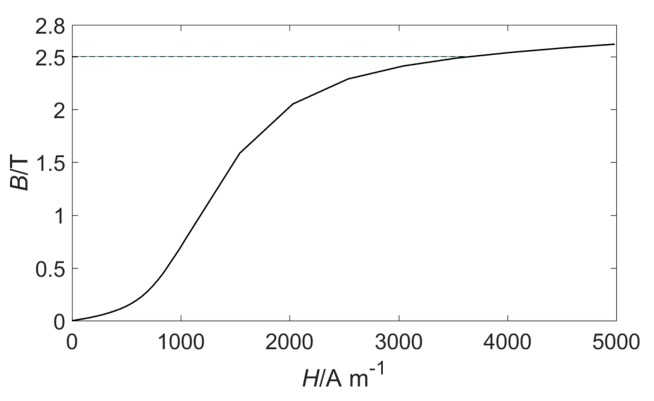
Magnetization curve of the 24 mm wire rope.

In order to ensure a good detection effect, the wire rope should be magnetized to a saturated state, and a sufficient magnetic leakage flux can be generated in the event of damage [4]. The magnetization curve of the wire rope with a diameter of 24 mm measured by a laboratory instrument is displayed in Figure 4. It can be seen from the figure that it is necessary to ensure that the magnetic flux density B of the wire rope is at least 2.5 T.

**Figure 5:**
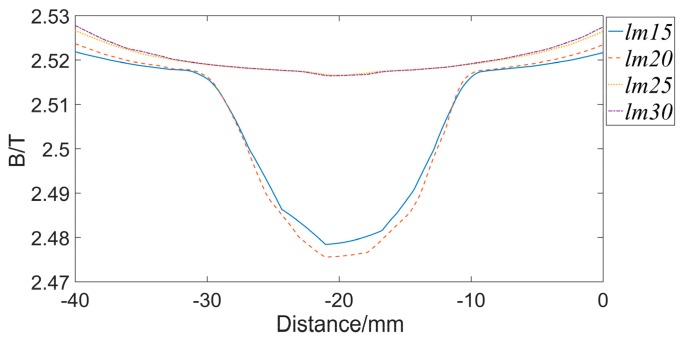
Magnetic flux density of wire ropes with different magnet lengths.

The length of the permanent magnet $l_{m}$ = 20 mm and the distance between the two magnetic poles $L_{m}$ = 160 mm, which are calculated by the Equations in Section 2.1. In order to achieve an ideal magnetization effect, set $l_{m}$ = 15 mm, 20 mm, 25 mm, and 30 mm, respectively, while other sizes remain unchanged. A three-dimensional model is established and simulated. Figure 5 presents the result. The abscissa represents the axial position of the wire rope, the 0 coordinate point is the center of the axial direction of the exciter, the 0 coordinate is also the axial center of the magnetized wire rope (the same below). The ordinate represents the magnetic flux density of the wire rope. When the $l_{m}$ is small, the wire rope can not be magnetized to saturation, especially in the middle position, the magnetic flux density of the wire rope is significantly lowered. Magnetic sensitive element is usually required to be placed here, so the performance of the detection will be affected. When $l_{m}$ increases, the magnetic flux density of the wire rope also rises. When $l_{m}$ = 25 mm, the magnetic flux density in the wire rope reaches 2.5T or more. However, if the $l_{m}$ continues to increase, the magnetic flux density of the wire rope does not change much. This happens because the wire rope has reached saturation. Therefore, taking $l_{m}$ = 25 mm is most suitable option.

**Figure 6:**
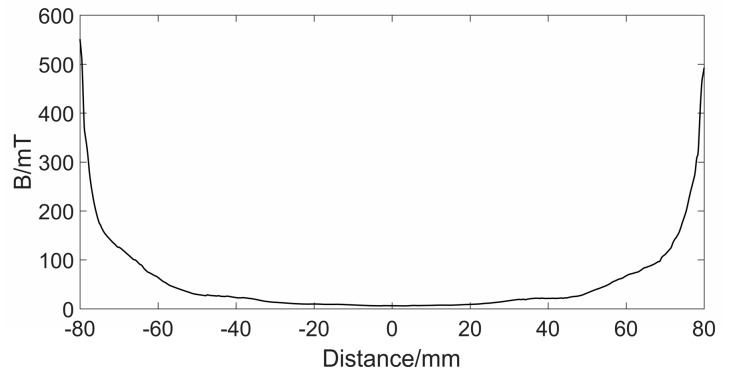
Magnetic flux density change from magnetic poles to the intermediate position of the exciter.

On the surface of the wire rope, the magnetic flux intensity gradually decreases from the two magnetic poles to the middle of the sensor, forming a transitional magnetization section, as displayed in Figure 6. If the distance between the poles is large enough, a uniform magnetization section with zero magnetic flux will be formed in the middle of the exciter. This will greatly facilitate the detection of magnetic leakage flux. However, from the rationality of the design, it is impossible to form an absolutely uniform magnetization section. Only an appropriate distance between magnetic poles can be set to form a relatively uniform magnetization section. If the magnetic pole spacing is too small, the magnetic flux will pass through the surface of the wire rope, which will affect the detection effect. Meanwhile, the overlong magnetic pole spacing will lead to larger weight of the sensor. Therefore, it is very important to take a suitable magnetic pole spacing.

**Figure 7:**
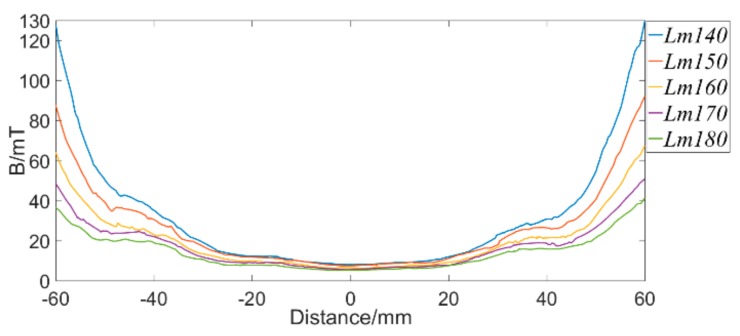
Magnetic flux density at intermediate positions of exciter with different $L_{m}$

**Figure 8:**
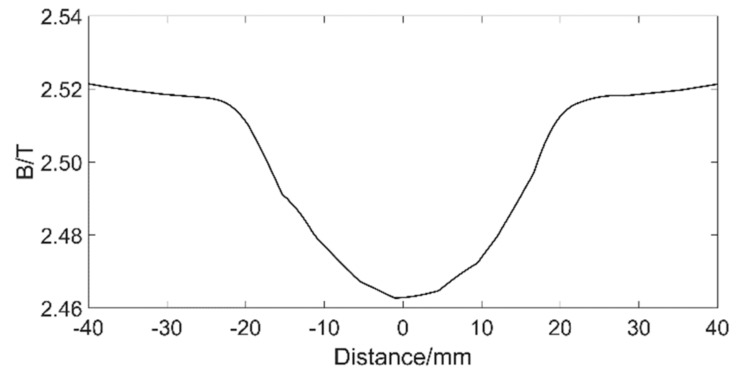
Magnetic flux density of the wire rope with $L_{m}$ = 180 mm.

Keeping $l_{m}$ = 25 mm and other sizes unchanged, set $L_{m}$ = 140 mm, 150 mm, 160 mm, 170 mm, and 180 mm, respectively. The simulation results are shown in Figure 7. From the graph, we can see that the uniform magnetization section becomes more and more obvious with the increase of $L_{m}$. Moreover, the magnetic flux density in the middle position is smaller and the fluctuation is smaller, indicating that the magnetic induction lines are more uniform. When damage occurs here, the MFL signal is affected by the original magnetic flux to a less extent. However, the length of the wire rope to be magnetized by the exciter becomes longer with the increase of $L_{m}$, resulting in part of the wire rope not reaching saturation. When $L_{m}$ = 180 mm, the magnetic flux density of the wire rope is shown in Figure 8. It can be seen that there is a drop in the middle position, which shows that the wire rope is not saturated. Therefore, in order to ensure a good magnetization effect, and to form a more uniform magnetization segments as much as possible, setting $L_{m}$ = 170 mm is the best choice.

### 3.2. Simulation of Magnetic Concentrator 

**Figure 9:**
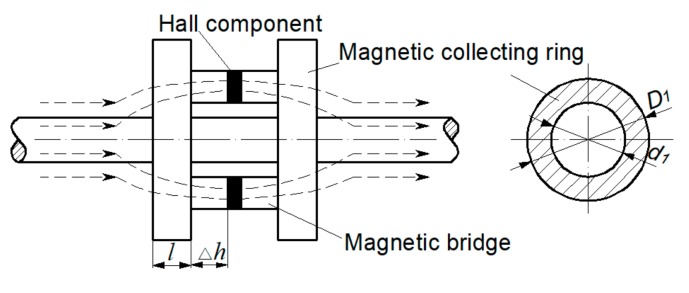
Structure and dimensions of the magnetic concentrator.

**Figure 10:**
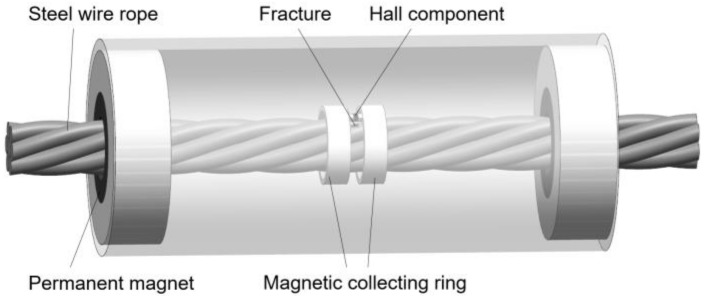
Overall simulation model.

The structure and dimensions of the magnetic concentrator [15] are shown in Figure 9. The concentrator is placed in the middle of the designed sensor, and it is composed of magnetic collecting rings and magnetic bridges, both of which are made of high magnetic permeability material, such as industrial pure iron, ink-repellent alloy, etc. When a magnetic leakage flux is generated, the magnetic collecting ring can gather the magnetic flux, and transmitted it to the magnetic ring on the other side through the magnetic bridge, and a neck-shaped detecting path is formed around the wire rope. The Hall component is placed on the bridge, ensuring that most of the leakage flux passing through the Hall component. In order to eliminate the noise of the magnetic leakage flux between the strands, the length of the magnetic collecting ring l should be one-half of the strand interval [14]. In this paper, the sensor is designed for 24 mm-diameter wire ropes, so l = 12 mm, Δh = 2 mm [14]. The outer diameters $D_{1}$ and inner diameters $d_{1}$ of the magnetic collecting ring will be determined according to the lift-off. The sensor is modeled according to the sizes above. Different parameters of the magnetic collecting ring are given in Table 1. The overall model is shown in Figure 10. The wire rope is replaced by seven steel strands as a simulation specimen, and a fracture of 2 mm × 2 mm × 2 mm is made on the specimen.

**Figure 11:**
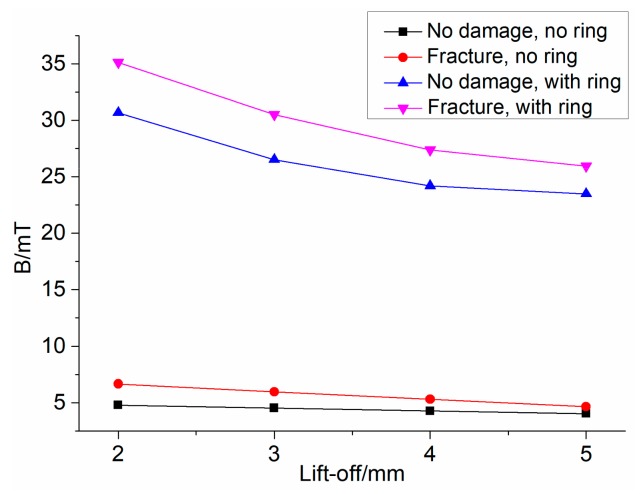
Magnetic flux density of Hall component under different lift-off.

The simulation is carried out with or without magnetic concentrators, respectively. The Hall component is located directly above the fracture. The magnetic flux density of the Hall component with different lift-off is displayed in Figure 11. In the figure, regular triangles and inverted triangles represent the case of a magnetic concentrator, and the square and circle illustrate no concentrator. It is apparent that the magnetic flux density of the Hall component is decreased with the increase of lift-off. In the absence of a magnetic concentrator, the magnetic flux density induced by the Hall component does not change much after the damage occurs. When there is a magnetic concentrator, the difference of magnetic flux density before and after the damage is more obvious. This difference is more easily detected by magnetic sensors, which indicates the good performance of the concentrator on collecting the MFL signal.

Moreover, it can be found that when the lift-off becomes larger, the difference of magnetic flux density becomes small regardless of the presence or absence of the magnetic concentrator. Therefore, the lift-off should be as small as possible. However, if the lift-off is too small, the slight change of the wire rope at the axial center of the sensor will have a great influence on the detection effect. In fact, many physical factors may cause the wire rope to fluctuate about 2–3 mm [21], so the lift-off cannot be set too small. As can be seen from Figure 11, when the distance is 4 mm, there is still a good resolution before and after the damage in the case of using a magnetic concentrator. Therefore, the lift-off is 4 mm in this paper, which means $D_{1}$ = 42 mm, $d_{1}$ = 32 mm.

**Figure 12:**
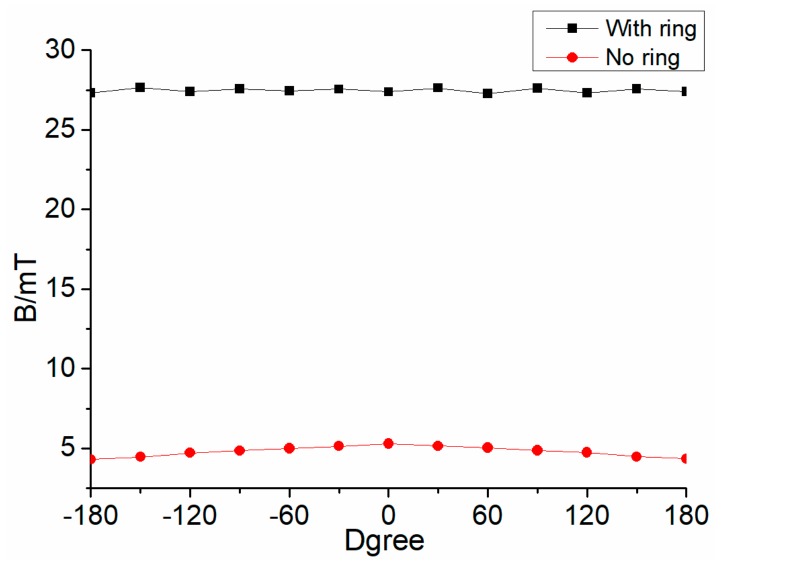
Magnetic flux density of the Hall component with different angles.

The detection effect of the magnetic concentrator is studied when the angle between the Hall component and the damage changed. When the Hall component is just above the damage and the damage and the Hall component are closest to each other, the angle is defined as a 0°; When the radial distance between the damage and the Hall component is the farthest, it is defined as 180°. Each angle change is 30°, and the lift-off is 4 mm. The simulation result is presented in Figure 12, with no magnetic concentrator, the magnetic flux density is the largest when the angle is 0°. The value becomes smaller gradually with the angle changes. When the angle is greater than 120°, the magnetic flux density of the Hall component is almost equal to the value with no damage, which shows that the Hall component has substantially failed to sense the magnetic leakage flux generated by the damage. When there is a concentrator, the magnetic flux density of the Hall component does not change much with angle changed, and the difference is basically within 0.4 mT. This shows that the magnetic concentrator has good performance on collecting MFL, and it is basically not affected by the angle between Hall element and damage. Overall, the collection of the magnetic leakage flux in the whole circumferential direction can be realized by the magnetic concentrator and a small number of Hall components, and the signal processing can be simplified.

## 4. Experiment and Discussion

### 4.1. Sensor Based on Magnetic Concentration Principle

**Figure 13:**
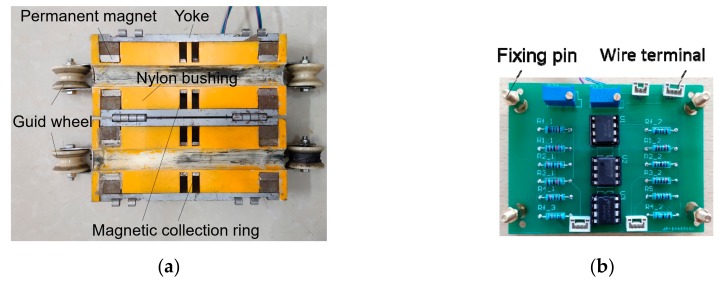
Prototype of the designed sensor: (**a**) Photographic view of the sensor; (**b**) Printed circuit board.

The configuration structure of the proposed sensor is illustrated in Figure 13a. When the sensor is combined, the diameter of the sensor is 94 mm and the total length is 220 mm. The whole structure is divided, which is convenient for installation. The ring-shaped NdFeB35 permanent magnet is employed for the magnetization of the wire rope, the magnetic properties of the NdFeB35 are presented in Table 2 The magnetization direction of the magnet is radial. The magnetic collecting ring is also made into a half ring. One bridge is set on each pair of half rings, and one Hall component is placed on each bridge. The material of the magnetic collecting ring and the yoke is industrial pure iron DT4. The permanent magnet, the yoke, and the magnetic collecting ring are embedded on the nylon bushing. When the sensor is combined, the two halves of the magnet are combined into a whole ring to magnetize steel wire rope, and the magnetic collecting ring is combined to collect a MFL. A printed circuit board (PCB), shown in Figure 13b, is designed to perform a pre-processing for the original signal. The Hall component and the PCB are connected by wire terminals. PCB supplies power to the Hall component. The Hall component has a static output voltage, so the PCB has a zeroing function. When there is no damage, making the output of the whole circuit is zero by adjusting the sliding rheostat. The PCB has a two-stage amplifying circuit with a magnification of 100 times, and the output voltage of the board is the sum of the outputs of the two Hall components.

**Figure 14:**
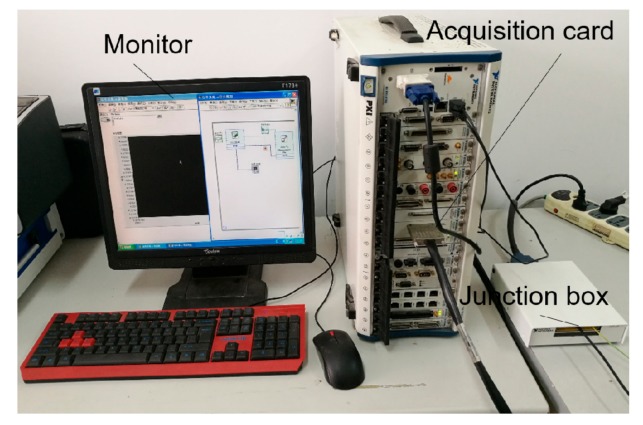
Data acquisition system.

Figure 14 shows the acquisition system. The output of the Hall component is processed by the PCB and then connected to the PXI-6281 integrated data acquisition card through NI’s SCB-68 signal junction box. The acquisition card has a programmable low-pass filter, which has good suppression ability for the frequency aliasing phenomenon during the signal acquisition process. The acquisition card can convert the voltage signal into a digital signal. The LabVIEW acquisition program is used to acquire the signal, and the signal is displayed in real time through a monitor.

### 4.2. Experimental Process

**Figure 15:**
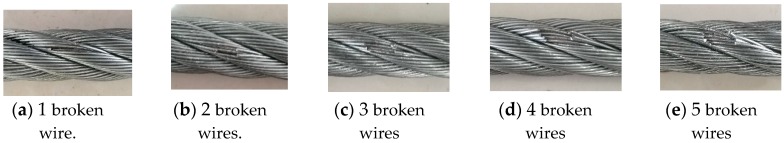
Broken wire damage.

A 6 × 24 + FC galvanized wire rope with a diameter of 24 mm is for broken wire detection, the wire diameter is 1.1 mm and the total length of the wire rope is 5350 mm. Five broken wires located in different circumferential positions are handcrafted on the surface of the wire rope. The distance between the broken wires is maintained at 250 mm, which is slightly more than the length of proposed sensor (220 mm). As displayed in Figure 15, the number of broken wires is 1 to 5 from the left to the right of the Figure, and the size of the fracture of each broken wire is 10 mm.

**Figure 16:**
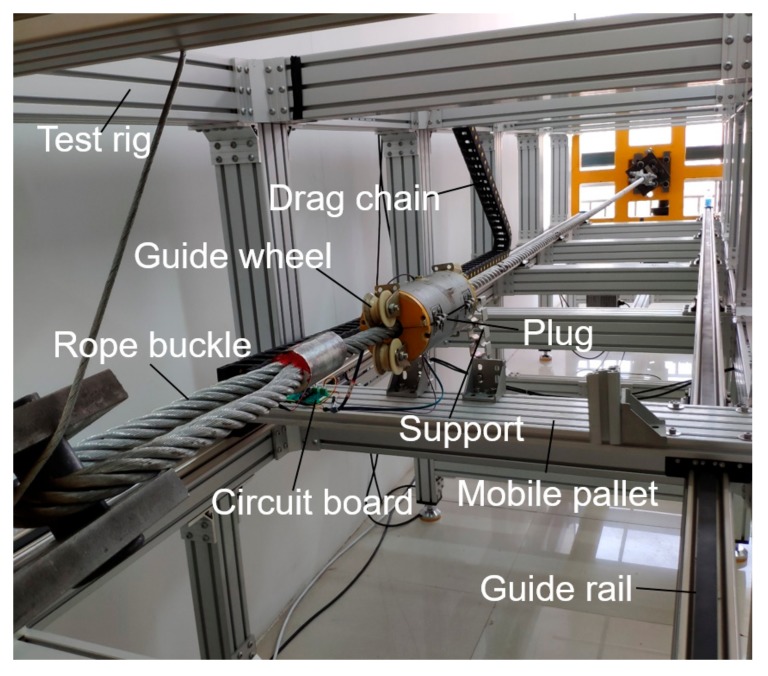
Test rig.

As shown in Figure 16, the wire rope is placed on the steel wire rope test rig for the inspection. There are rope buckles at both ends of the wire rope, which are fixed at both ends of the test rig. The frame of the whole rig is made of aluminium alloy, which can avoid affecting the MFL signals. The sensor is mounted on the wire rope and locked by two plugs, and then fixed by two aluminium alloy brackets on the mobile pallet. The outer end of the sensor has a guide wheel to ensure that the wire rope is at the axial center of the sensor. The PCB is fixed on the pallet by four fixing pins (Figure 13b), and the output signal of the PCB is transmitted to the junction box by the drag chain cable. The drag chain ensures that the transmission line is not entangled and scattered, and the chain moves along with the pallet to ensure the stability of PCB. Mobile pallet are driven by two guide rails, and the damage detection of the wire rope can be realized by the movement of the sensor. During the experiment, the moving speed of the sensor is 0.25 m/s.

### 4.3. Experimental Results and Analysis

**Figure 17:**
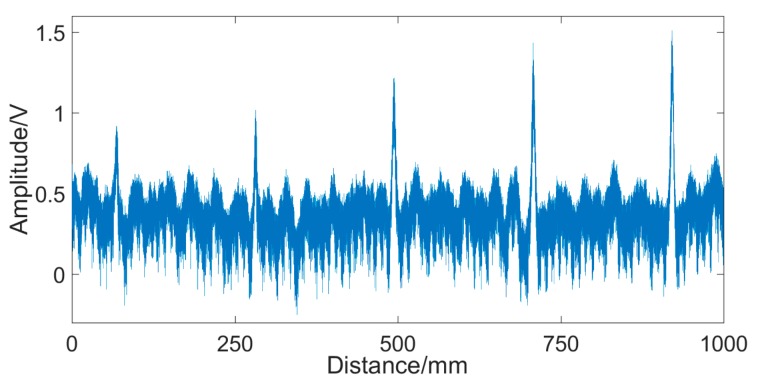
Signal of broken wire damage.

Figure 17 shows the experimental results obtained from the wire rope with a row of broken wires. The abscissa represents the moving distance of the sensor on the wire rope, and the ordinate is the voltage amplitude. It can be seen clearly that there are five saltations in the signal. Five saltations correspond to five broken wires, which indicates that the designed sensor can identify 1–5 external broken wires of the 24 mm-diameter wire rope.

The original signal has noise generated by the circuit and the external environment. In order to improve the signal-to-noise ratio (SNR) of the signal, the wavelet transform is used to denoise the signal [22,23,24].

**Figure 18:**
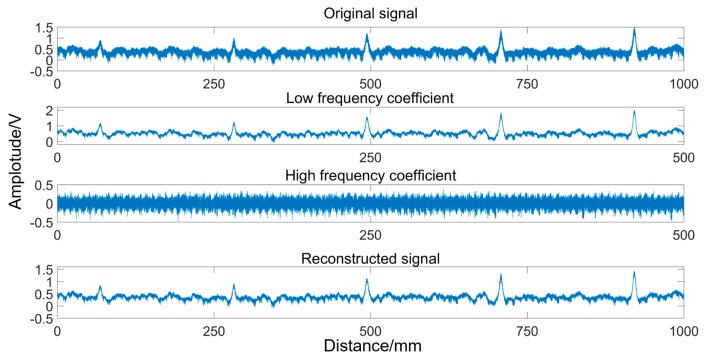
Denoising by DWT.

The third-order Daubechies wavelet is selected as the mother wavelet, and the one-dimensional discrete wavelet transform is performed on the original signal in the Matlab environment. Figure 18 presents the process of denoising. It can be seen that the low frequency coefficient preserves the damage characteristics of the original signal, and the noise is mainly concentrated in the high frequency part. The signal is reconstructed using the low frequency coefficients, and the high frequency coefficients portion of the signal is discarded. Obviously, the reconstructed signal effectively removes most of the noise and completely preserves the characteristics of the broken wire signal, and the SNR of the MFL signal is significantly improved.

### 4.4. Comment and Discussion

The above results show that the proposed sensor can recognize the external broken wires of 24 mm-diameter wire rope. However, we only tested the detection effect under a specific fracture length. We have not studied the smallest fracture that the sensor can identify due to the difficulty of sample preparation. Meanwhile, the distance between broken wires remains constant. The nearest distance between two broken wires that the sensor can recognize is also an important portion we will focus on in the next step.

## 5. Conclusions

In order to meet the requirements of damage detection of steel wire ropes in various working conditions, an innovative sensor for the detection of broken wires based on magnetic concentrating principle is proposed. 

To make the excitation more uniform and the wire rope more easily magnetized to saturation, a CMPME is used in this design. The influence of its two parameters $l_{m}$ and $L_{m}$ on the excitation effect is analyzed through the finite element method, which provides a basis for the optimization of the structure of the permanent magnet exciter.

The traditional Hall sensor array is replaced by a magnetic concentrator. The magnetic concentrator can achieve a comprehensive collection of the magnetic flux leakage (MFL) compared with separate Hall component, and the use of Hall components can be greatly reduced, which simplifies the subsequent signal processing.

The designed sensor is examined through an experiment. The results show that 1–5 external broken wires can be obviously recognized by the sensor, which indicates the superior performance of the designed sensor on faults inspection of steel wire ropes.

Although the sensor proposed in this paper has good performance on inspecting external broken wires, we haven’t researched its detectable minimum damage and axial resolution yet. Meanwhile, it has not been tested for other damages of wire ropes such as internal broken wires and wear. Furthermore, if the sensor can detect the signals of these damages, it is necessary to provide an appropriate method for fault classification in future work.

## Figures and Tables

**Table 1:** Outer diameter and inner diameter of the magnetic ring under different lift-off.

Lift-off	D1	d1
2 mm	38 mm	28 mm
3 mm	40 mm	30 mm
4 mm	42 mm	32 mm
5 mm	44 mm	34 mm

**Table 2:** Properties of the NdFeB35 permanent magnet.

Brand	Br	Hcb	maxBH
N35	1170–1230 mT	10.7–12.0 kOe	264–288 kJ/m^3^
	11.7–12.3 kGs	852–955 kA/m	33–36 MGOe
